# The impact of stigma and socioeconomic barriers on HIV treatment adherence in Conakry, Republic of Guinea: a gender perspective

**DOI:** 10.11604/pamj.2025.52.180.46015

**Published:** 2025-12-23

**Authors:** Annamaria Doro Altan, Fausto Ciccacci, Marie Rose Tounkara, Adama Bountouraby Sylla, Claudia Mosconi, Carolina De Santo, Mariagrazia Cicala, Cristina Cannelli, Stefano Orlando

**Affiliations:** 1 Link Campus University, Rome, Italy,; 2 Department of Biomedicine and Prevention, University of Rome Tor Vergata, Rome, Italy,; 3 Dream Program, Community of Sant´Egidio, Conakry, Republic of Guinea,; 4 Dream Program, Community of Sant´Egidio, Rome, Italy

**Keywords:** HIV treatment adherence, stigma and socioeconomic barriers, gender differences in healthcare

## Abstract

**Introduction:**

HIV infection remains a major global health challenge, with sub-Saharan Africa being the region most heavily affected by the pandemic. Despite increased access to antiretroviral therapy (ART), adherence and retention in care remain problematic in many parts of West Africa, including the Republic of Guinea. Stigma, socioeconomic challenges, and healthcare access are key factors influencing treatment outcomes. This study aims to explore the determinants of ART adherence and the impact of stigma among people living with HIV (PLHIV) in Conakry, with a specific focus on gender differences.

**Methods:**

a cross-sectional study was conducted in four Dream Centers in Conakry, Coyah, and Dubreka, Guinea. Data were collected using a structured questionnaire covering participants' relationship with healthcare services, adherence to treatment, experiences of stigma, and sociodemographic characteristics. Adherence was measured using the Morisky Medication Adherence Scale (MMAS-8), and stigma was assessed using a modified HIV Stigma Scale. Descriptive and multivariate analyses were performed to identify factors associated with treatment adherence and discontinuation.

**Results:**

during the study period, 472 patients met the inclusion criteria, of whom 70 refused to participate; a total of 402 participants were included, with 330 (82%) actively engaged in care and 72 (18%) re-engaging after treatment interruption. The median age was 32 years (IQR: 27-40), with 82% female and 18% male participants. Education levels were 37% illiterate and 63% educated, with 91% employed and 9.5% unemployed. Among participants, 52% had children, and the median travel time to the Dream center was 1 hour (IQR: 0.75-2). High stigma levels were reported by 42%. Patients who were lost to follow-up and later re-engaged lived farther from the clinical center (median: 2 vs. 1 hour, OR 1.18 [1.00-1.39], p= 0.038) and had lower adherence rates (78% vs. 32%, OR 0.14 [0.07-0.24], p < 0.001). Patients with lower adherence rates were more often in therapy for over one year (82% vs. 96%, COR 0.19 [0.07-0.43], p < 0.001). Conversely, those with higher adherence rates reported greater trust in treatment (99.6% vs. 77%, COR 42 [8.65-757], p < 0.001), were more likely to live with children (59% vs. 41%, COR 1.93 [1.23-3.04], p = 0.004), and were less likely to report high stigma scores (34% vs. 55%, OR 0.55 [0.34-0.87], p = 0.011). Among men, stigma was the only significant factor for low adherence (OR: 0.32, 95% CI: 0.10-1.00, p= 0.05), while for women, higher adherence was associated with shorter time in care, higher trust in treatment, and having children (AOR 0.22 [0.08-0.52], p = 0.001; AOR 38.5 [7.80-698], p < 0.001; AOR 2.09 [1.28-3.46], p = 0.004).

**Conclusion:**

stigma and healthcare access are critical barriers to ART adherence among PLHIV in Conakry, Guinea. Gender-specific differences suggest that tailored interventions are needed to address these unique challenges. Reducing stigma, ensuring consistent follow-up, and fostering trust in healthcare providers are essential strategies to improve adherence and treatment outcomes. Addressing these barriers can enhance the quality of care and support viral suppression among PLHIV in Guinea.

## Introduction

HIV infection continues to represent a global challenge, with approximately 39.9 million people living with the virus. According to UNAIDS data, sub-Saharan Africa remains the epicenter of the pandemic, accounting for around 64.9% of all cases [1]. West and Central Africa continue to face significant barriers in combating the infection, with only 76% of people living with HIV on treatment, and just 70% of those achieving viral suppression [2]. According to the latest available UNAIDS data, there are approximately 130,000 people living with HIV in the Republic of Guinea, with a prevalence rate of about 1.3% among the population aged 15 to 49 years. Despite the availability of antiretroviral therapy (ART), many individuals struggle to access treatment, and only 88,000 were on therapy in 2023, representing 67.9% of the infected population [3]. Poor adherence among HIV patients in West Africa is influenced by multiple interconnected factors, posing significant challenges to effective treatment. Research has identified that difficulties in accessing healthcare, poverty, stigma, lack of social support, mental health issues, substance abuse, side effects of medications, and complex treatment regimens all negatively affect adherence to HIV treatment in the region [4]. The high prevalence of antiretroviral drug resistance among treatment-naive patients in the Republic of Guinea and Niger further suggests adherence issues and the spread of resistant variants, complicating treatment efforts [5]. Healthcare challenges, such as the lack of diagnostic tests, also negatively impact adherence, limiting the ability to properly manage and monitor patients [6]. In addition, the lack of awareness of serological status and poor management of comorbidities contribute to poor adherence among HIV patients [7]. Among the other factors, UNAIDS has highlighted the critical role of stigma in influencing treatment adherence among people living with HIV [8]. In summary, poor adherence to HIV treatment can be influenced by a complex mix of barriers, including challenges in accessing healthcare, socioeconomic difficulties, mental health issues, stigma, and obstacles related to treatment itself. The aim of our study is to analyze the factors associated with treatment discontinuation and the perception of stigma among HIV patients in the regions of Conakry and Kindia, Republic of Guinea, with a focus on gender differences.

## Methods

**Study design and setting:** we conducted a cross-sectional study using a structured questionnaire. The research was carried out at the DREAM Centers in Conakry (Dixinn, Conakry region), the Soumaya DREAM Center (Coyah, Kindia region), and the Dubreka DREAM Center (Kindia region). All sites are located in the Republic of Guinea, within the geographical region of Basse Guinea. The DREAM program is a public health initiative active in ten African countries, providing HIV care and other health services [9-13].

**Participants and sampling method:** the study sample included people living with HIV (PLHIV) aged 18 years or older who were currently in care, and who had been attending the DREAM Centers for no more than five years, including those who had interrupted treatment and reopened their clinical records. On randomly selected days, trained interviewers approached eligible patients in the clinic waiting rooms. In addition, patients who re-engaged in care after an interruption were invited to participate. Exclusion criteria included being under 18 years old or being enrolled in care for more than five years.

**Sample size estimation:** the minimum sample size was calculated based on a 95% confidence level, an estimated proportion of 25% of poor adherence (as indicated by the Morisky scale), and a 5% margin of error, resulting in a minimum sample size of 288 participants. An additional 20% was added to account for potential errors, leading to a final sample size of approximately 350 participants.

**Data collection and instruments:** data were collected using a structured questionnaire divided into four sections: relationship with health services, treatment adherence, stigma experience, and sociodemographic characteristics. The Morisky Medication Adherence Scale (MMAS-8) was used to assess adherence, with scores categorized as high (8), medium (6-7), or low (<6) [14,15]. Stigma was measured with a simplified version of the HIV Stigma Scale (HSS), culturally adapted in collaboration with clinical staff and peer educators living with HIV [16]. Ten trained interviewers (patients or psychosocial staff not directly involved in care) administered the questionnaire after obtaining written informed consent. The tool was piloted and adjusted for clarity and cultural appropriateness. Data were entered using Google Forms, with each form assigned a unique ID code.

**Measures and variables:** the primary outcomes were treatment status (currently in care vs. re-engaged after loss to follow-up), and Adherence: MMAS-8 score <7 (low) vs. ≥7 (high). Explanatory variables included age, sex, time on ART (<1 year vs. >1 year), travel time to clinic (in hours), education level, employment status, living with children (yes/no), and stigma level (categorized by median score).

**Statistical analysis:** data were analyzed using R software version 4.3.1 (2023-06-16). Descriptive statistics include frequencies and percentages for categorical variables and medians with interquartile ranges for continuous variables. Associations with outcomes were assessed using univariate logistic regression to calculate crude odds ratios (CORs), followed by multivariate logistic regression for adjusted odds ratios (AORs), with 95% confidence intervals. Analyses were stratified by gender to assess sex-specific determinants of adherence.

**Ethics statement:** written informed consent was obtained from all participants, and confidentiality was maintained throughout the study. Questionnaires were anonymous, and consent forms were stored separately from the questionnaires. Authorization was obtained from the National Ethics Committee for Health Research in Guinea (number 202/CNERS/23, 15 November 2023).

## Results

**Participant characteristics:** during the study period, 472 patients met the inclusion criteria and were invited to join the study. Of these, 70 refused to participate due to reasons such as lack of time or fear of encountering acquaintances. A total of 402 participants were included in the study, with 330 (82%) actively engaged in care at the DREAM centers and 72 (18%) re-engaging after a period of treatment interruption. Table 1 presents the results of the analysis for the entire study population. The median age of participants was 32 years (IQR: 27-40). The majority of participants were female (n= 328, 82%) compared to male (n= 74, 18%). Education level was categorized as illiterate (n = 149, 37%) or educated (including primary, secondary, and university education) (n = 253, 63%). Employment status was categorized as employed (n= 363, 91%) or unemployed (n = 38, 9.5%), and 52% (n= 209) of participants had children. The median travel time to the DREAM center was 1 hour (IQR: 0.75-2). Stigma level was evaluated to assess its impact on treatment adherence, with 42% (n = 170) reporting high levels of stigma.

**Table 1:** descriptive characteristics of the entire sample by participant status and adherence levels

	Entire sample	Descriptive by participant status					Descriptive by Morisky							
**Characteristic**	**Overall, N = 4021**	**Continously treated N = 3301**	**Re-engaged N = 721**	**OR^2^**	**95% CI^2^**	**P-value**	**< 7 N = 163^3^**	**> 7N = 239^3^**	**COR^2^**	**95% CI^2^**	**P-value**	**AOR^2^**	**95% CI^2^**	**P-value**
Age (years)	32 (27-40)	32 (26-40)	33 (28-39)	1.01	0.98-1.03	0.6	32 (28-40)	32 (26-40)	1.00	0.98-1.02	0.8			
**Sex**														
F	328 (82%)	267 (81%)	61 (85%)	-	-		132 (81%)	196 (82%)	-	-				
M	74 (18%)	63 (19%)	11 (15%)	0.76	0.36-1.49	0.5	31 (19%)	43 (18%)	0.93	0.56-1.57	0.8			
**Education status**														
Educated	253 (63%)	212 (64%)	41 (57%)	-	-		96 (59%)	157 (66%)	-	-		-	-	
Illiterate	149 (37%)	118 (36%)	31 (43%)	1.36	0.80-2.28	0.2	67 (41%)	82 (34%)	0.75	0.50-1.13	0.2	0.75	0.47-1.18	0.2
**Profession**														
Unemployed	38 (9.5%)	34 (10%)	4 (5.6%)	-	-		17 (10%)	21 (8.8%)	-	-				
Employed	363 (91%)	296 (90%)	67 (94%)	1.92	0.74-6.61	0.2	145 (90%)	218 (91%)	1.22	0.61-2.38	0.6			
Unknown	1	0	1	;			1	0						
**Time since enrollment at Dream**														
< 1 year	51 (13%)	46 (14%)	5 (6.9%)	-	-		7 (4.3%)	44 (18%)	-	-		-	-	
> 1 year	351 (87%)	284 (86%)	67 (93%)	2.17	0.91-6.44	0.11	156 (96%)	195 (82%)	0.20	0.08-0.43	<0.001	0.19	0.07-0.43	<0.001
Distance from dream center (hours)	1.00 (0.75-2.00)	1.00 (0.75-2.00)	2.00 (1.00-3.00)	1.18	1.00-1.39	0.038	1.50 (0.83-2.50)	1.00 (0.75-2.00)	0.89	0.77-1.02	0.10			
**Trust in the treatment**														
N	38 (9.5%)	27 (8.2%)	11 (15%)	-	-		37 (23%)	1 (0.4%)	-	-		-	-	
Y	364 (91%)	303 (92%)	61 (85%)	0.49	0.24-1.09	0.067	126 (77%)	238 (99.6%)	69.9	14.9-1,248	<0.001	42.0	8.65-757	<0.001
**Living with children**														
N	193 (48%)	151 (46%)	42 (58%)	-	-		96 (59%)	97 (41%)	-	-		-	-	
Y	209 (52%)	179 (54%)	30 (42%)	0.60	0.36-1.01	0.054	67 (41%)	142 (59%)	2.10	1.40-3.15	<0.001	1.93	1.23-3.04	0.004
**Stigma score**														
< 5.5	232 (58%)	190 (58%)	42 (58%)	-	-		74 (45%)	158 (66%)	-	-		-	-	
**> 5.5**	170 (42%)	140 (42%)	30 (42%)	0.97	0.57-1.62	>0.9	89 (55%)	81 (34%)	0.43	0.28-0.64	<0.001	0.55	0.34-0.87	0.011
**Morisky score**														
< 7	163 (41%)	107 (32%)	56 (78%)	-	-									
> 7	239 (59%)	223 (68%)	16 (22%)	0.14	0.07-0.24	<0.001								
**Participant status**														
Continously treated							107 (66%)	223 (93%)	-	-				
Re-engaged							56 (34%)	16 (6.7%)	0.14	0.07-0.24	<0.001			

1Median (IQR); n (%); 2OR : odds ratio, CI: confidence interval; 3n (%); Median (IQR)

**Factors associated with treatment discontinuation:** patients who were lost to follow-up and later re-engaged in care lived, on average, farther from the clinical center (median: 2 vs. 1 hour, OR 1.18 [1.00-1.39], p = 0.038) and reported lower adherence rates (78% vs. 32%, OR 0.14 [0.07-0.24], p < 0.001).

**Factors associated with adherence to antiretroviral therapy:** in the multivariate analysis, adherence level was significantly associated with several factors: time in therapy, trust in treatment, living with children, and stigma score. Patients with lower adherence rates were more often in therapy for over one year (82% vs. 96%, COR 0.19 [0.07-0.43], p < 0.001). Conversely, patients with higher adherence rates reported greater trust in treatment (99.6% vs. 77%, COR 42 [8.65-757], p < 0.001), were more likely to live with children (59% vs. 41%, COR 1.93 [1.23-3.04], p= 0.004), and were less likely to report high stigma scores (34% vs. 55%, OR 0.55 [0.34-0.87], p= 0.011).

**Gender differences in adherence determinants:** the multivariate analysis, presented in Table 2 and Table 3, identifies distinct factors associated with poor adherence among male and female participants. For male participants, the only factor showing a marginally statistically significant association with low adherence was a stigma score higher than 5.5 (OR: 0.32, 95% CI: 0.10-1.00, p = 0.05). In contrast, for female participants, several factors were independently associated with low adherence, including time in care, trust in treatment, and living without children. Female patients with low adherence were more likely to have been in treatment for over one year (95% vs. 80%, AOR 0.22 [0.08-0.52], p = 0.001), to report lower trust in antiretroviral treatment (77% vs. 99%, AOR 38.5 [7.80-698], p < 0.001), and to live without children (60% vs. 40%, AOR 2.09 [1.28-3.46], p= 0.004).

**Table 2:** descriptive characteristics and predictors of adherence among male participants

	Male patients	Descriptive by participant status					Descriptive by Morisky							
**Characteristic**	**Overall**-N = 741	**Continously treated, N= 631**	**Re-engaged, N = 111**	**OR ^2^**	**95% CI ^2^**	**P-value**	**< 7 N= 313**	**> 7 N = 433**	**COR ^2^**	**95% CI^2^**	**P-value**	**AOR^2^**	**95% CI^2^**	**P-value**
Age (years)	40 (32-48)	41 (33-48)	38 (31-56)	1.01	0.96-1.07	0.6	38 (34-46)	42 (32-51)	1.02	0.98-1.07	0.3			
**Education status**														
Educated	59 (80%)	51 (81%)	8 (73%)	-	-		22 (71%)	37 (86%)	-	-		-	-	
Illiterate	15 (20%)	12 (19%)	3 (27%)	1.59	0.31-6.50	0.5	9 (29%)	6 (14%)	0.40	0.12-1.25	0.12	0.40	0.11-1.41	0.2
Profession					;									
Unemployed	15 (20%)	14 (22%)	1 (9.1%)	-	-		11 (35%)	4 (9.3%)	-	-		-	-	
Employed	59 (80%)	49 (78%)	10 (91%)	2.86	0.48-54.7	0.3	20 (65%)	39 (91%)	5.36	1.61-21.4	0.009	3.05	0.78-13.3	0.12
**Time since enrollment at Dream**														
< 1 year	5 (6.8%)	5 (7.9%)	0 (0%)	-	-		0 (0%)	5 (12%)	-	-				
> 1 year	69 (93%)	58 (92%)	11 (100%)	NA	NA	NA	31 (100%)	38 (88%)	0.00		>0.9			
Distance from DREAM center (hours)	1.50 (1.00-2.00)	1.50 (1.00-2.00)	2.00 (1.50-3.00)	1.16	0.79-1.59	0.4	1.50 (1.00-2.00)	1.50 (1.00-2.00)	0.97	0.72-1.30	0.8			
**Trust in the treatment**														
N	6 (8.1%)	5 (7.9%)	1 (9.1%)	-	-		6 (19%)	0 (0%)	-	-				
Y	68 (92%)	58 (92%)	10 (91%)	0.86	0.12-17.4	0.9	25 (81%)	43 (100%)	NA	NA	>0.9			
**Living with children**														
N	35 (47%)	29 (46%)	6 (55%)	-	-		17 (55%)	18 (42%)	-	-				
Y	39 (53%)	34 (54%)	5 (45%)	0.71	0.19-2.59	0.6	14 (45%)	25 (58%)	1.69	0.67-4.34	0.3			
**Stigma score**														
< 5.5	49 (66%)	39 (62%)	10 (91%)	-	-		15 (48%)	34 (79%)	-	-		-	-	
> 5.5	25 (34%)	24 (38%)	1 (9.1%)	0.16	0.01-0.93	0.093	16 (52%)	9 (21%)	0.25	0.09-0.67	**0.007**	0.32	0.10-1.00	0.050
**Morisky score**														
< 7	31 (42%)	22 (35%)	9 (82%)	-	-									
> 7	43 (58%)	41 (65%)	2 (18%)	0.12	0.02-0.51	**0.010**								
Participant status														
Continously treated							22 (71%)	41 (95%)	-	-				
Re-engaged							9 (29%)	2 (4.7%)	0.12	0.02-0.51	**0.010**			

1Median (IQR); n (%); 2OR: Odds Ratio, CI: Confidence Interval; 3n (%); Median (IQR)

**Table 3:** descriptive characteristics and predictors of adherence among female participants

	Female patients	Descriptive by Participant status					Descriptive by Morisky							
**Characteristic**	**Overall N = 328^1^**	**Continously treated N = 267^1^**	**Re-engaged N = 61^1^**	**OR^2^**	**95% CI^2^**	**P-value**	**< 7 N = 132^3^**	**> 7 N = 196^3^**	**OR ^2^**	**95% CI^2^**	**P-value**	**OR^2^**	**95% CI^2^**	**P-value**
Age (years)	30 (25-38)	30 (25-38)	32 (28-38)	1.01	0.98-1.04	0.5	31 (27-38)	30 (25-37)	1.00	0.97-1.02	0.8			
**Education status**														
Educated	194 (59%)	161 (60%)	33 (54%)	-	-		74 (56%)	120 (61%)	-	-				
Illiterate	134 (41%)	106 (40%)	28 (46%)	1.29	0.73-2.26	0.4	58 (44%)	76 (39%)	0.81	0.52-1.27	0.4			
Profession														
Unemployed	23 (7.0%)	20 (7.5%)	3 (5.0%)	-	-		6 (4.6%)	17 (8.7%)	-	-				
Employed	304 (93%)	247 (93%)	57 (95%)	1.54	0.51-6.69	0.5	125 (95%)	179 (91%)	0.51	0.18-1.25	0.2			
Unknown	1	0	1				1	0						
**Time since enrollment at dream**														
< 1 year	46 (14%)	41 (15%)	5 (8.2%)	-	-		7 (5.3%)	39 (20%)	-	-		-	-	
> 1 year	282 (86%)	226 (85%)	56 (92%)	2.03	0.83-6.09	0.2	125 (95%)	157 (80%)	0.23	0.09-0.49	<0.001	0.22	0.08-0.52	0.001
Distance from dream center (hours)	1.00 (0.75-2.00)	1.00 (0.75-2.00)	1.75 (1.00-3.00)	1.20	0.99-1.44	0.052	1.50 (0.75-2.52)	1.00 (0.75-2.00)	0.87	0.73-1.02	0.080			
**Trust in the treatment**														
N	32 (9.8%)	22 (8.2%)	10 (16%)	-	-		31 (23%)	1 (0.5%)	-	-		-	-	
Y	296 (90%)	245 (92%)	51 (84%)	0.46	0.21-1.06	0.058	101 (77%)	195 (99%)	59.9	12.6-1,073	<0.001	38.5	7.80-698	<0.001
**Living with children**														
N	158 (48%)	122 (46%)	36 (59%)	-	-		79 (60%)	79 (40%)	-	-		-	-	
Y	170 (52%)	145 (54%)	25 (41%)	0.58	0.33-1.02	0.062	53 (40%)	117 (60%)	2.21	1.41-3.48	<0.001	2.09	1.28-3.46	**0.004**
**Stigma score**														
< 5.5	183 (56%)	151 (57%)	32 (52%)	-	-		59 (45%)	124 (63%)	-	-		-	-	
> 5.5	145 (44%)	116 (43%)	29 (48%)	1.18	0.67-2.06	0.6	73 (55%)	72 (37%)	0.47	0.30-0.73	<0.001	0.62	0.37-1.04	0.068
**Morisky score**														
< 7	132 (40%)	85 (32%)	47 (77%)	-	-									
> 7	196 (60%)	182 (68%)	14 (23%)	0.14	0.07-0.26	<0.001								
Participant status														
Continously treated							85 (64%)	182 (93%)	-	-				
Re-engaged							47 (36%)	14 (7.1%)	0.14	0.07-0.26	<0.001			

1Median (IQR); n (%);2OR: odds ratio, CI: confidence interval; 3n (%); Median (IQR)

## Discussion

Our study examined barriers to treatment adherence among 402 people living with HIV in Conakry, Guinea, with a focus on gender differences. Stigma emerged as a critical barrier, especially among male participants, where perceived stigma was marginally associated with low adherence, suggesting specific challenges related to healthcare access and social perception. Disengagement from care resulted from the distance to the health center, stressing the importance of geographical accessibility for health facilities. For female participants, adherence was influenced by multiple factors, including duration of care, trust in treatment, and family responsibilities. Women with low adherence were more often in therapy for over a year and less likely to report trust in antiretroviral treatment, emphasizing the importance of long-term engagement and trust-building initiatives. Interestingly, women living with children were more likely to demonstrate higher adherence, possibly indicating that family responsibilities act as a motivating factor to remain in care. Overall, our findings highlight that trust in treatment is a strong predictor of adherence across both genders, reinforcing the need for psychosocial support tailored to gender-specific challenges. Moreover, addressing stigma among men and focusing on building sustained trust among women could help improve adherence outcomes in this population. Our findings align with existing literature, which emphasizes stigma as a critical barrier to treatment adherence among people living with HIV in sub-Saharan Africa. UNAIDS and Garollo *et al*. both identified stigma as a significant impediment to effective adherence to ART, which is in line with our results that demonstrate its powerful impact, especially among male participants [4,8]. Adding to this, many authors similarly noted that barriers in healthcare access severely hinder adherence-a finding that supports our observation that physical absence from health centers correlates strongly with poor adherence [6,17]. Breton *et al*. have found an elevated percentage (50%) of pregnant women not willing to disclose their HIV status at a hospital facility in Conakry, which was associated with difficulty in talking about HIV [18].

While previous studies, including Albus *et al*., suggest that socioeconomic disadvantages like unemployment and low education levels are substantial predictors of non-adherence [7], our multivariable analysis found that these factors were overshadowed by stigma. These findings suggest that stigma may exacerbate or even drive the effects of economic and educational constraints on treatment engagement, indicating a compounded challenge for patients facing multiple types of disadvantages. Gender differences, evident in our study, are also reflected in broader research. Charpentier *et al*. for instance, documented varied treatment outcomes between men and women, suggesting that family responsibilities might provide women with additional motivation to adhere to ART [5]. Similarly, a systematic review identified stigma, discrimination, and insufficient social support as primary adherence barriers across sub-Saharan Africa [19]. These findings align with our own results, highlighting that stigma could exert a particularly strong influence on male adherence. Further supporting this, Luke and Uzosike, in a study on adherence in Rivers State, Nigeria, reported that stigma, financial hardships, and lack of spousal support were significant obstacles-adding a contextual layer to our observations on gender-specific adherence challenges [20].

The gender-specific challenges documented by Cornell *et al*. also underscore critical disparities, showing that men often start ART at a more advanced disease stage and experience higher mortality rates than women [21]. This aligns with our findings that men encounter greater challenges related to stigma and access to care. Muula *et al*. further elaborate that, although more women participate in HAART programs than men in Southern Africa, they still face unique challenges, particularly due to social vulnerability and stigma [22]. Together, these studies suggest that while women may have greater access to ART, their adherence is influenced by different factors than men, often linked to caregiving roles and support networks. In our study, these gender differences underscore a recurring theme: men and women face distinct adherence challenges in ART, with women, for instance, more likely to adhere if they have children. This finding suggests that social roles and family responsibilities may serve as additional motivators for adherence among women, while men may require different forms of support. Our findings reinforce the need for targeted interventions that address these gender-specific barriers, such as enhancing men´s engagement in healthcare and bolstering social support systems for women to foster sustained adherence across populations as we already studied in other African contexts [23-25].

The findings highlight the importance of addressing stigma in HIV care to improve adherence, particularly for men. Reducing stigma and improving healthcare access, including consistent follow-up and proximity to health centers, are crucial for supporting adherence for all genders. Policies should focus on community-based stigma reduction and healthcare access for PLHIV. Healthcare providers must build patient trust and provide targeted gender-specific support, such as childcare assistance for women and overcoming education barriers. HIV treatment guidelines should address stigma and consider differentiated adherence support based on gender. The main limitations of this study include its cross-sectional nature, which limits the ability to establish causal relationships. Additionally, the reliance on self-reported data may introduce recall bias, particularly in the assessment of adherence and stigma levels. The sample, although representative of the DREAM centers in Conakry, may not fully capture the experiences of PLHIV in more remote areas of Guinea. Future studies should consider a longitudinal design and include participants from diverse geographical settings to enhance the generalizability of findings.

## Conclusion

In conclusion, our study underscores the critical impact of stigma and healthcare accessibility on adherence to HIV treatment among PLHIV in Conakry, Guinea. The identification of gender-specific barriers emphasizes the need for tailored interventions to address the distinct challenges faced by male and female patients. Key strategies for improving treatment outcomes and achieving viral suppression among PLHIV include reducing stigma, ensuring consistent follow-up, increasing the availability of on-the-ground health facilities, and building trust in healthcare providers.

### What is known about this topic

- Stigma, socioeconomic challenges, and healthcare access are recognized as critical barriers to adherence to antiretroviral therapy (ART) among people living with HIV (PLHIV) in sub-Saharan Africa, including Guinea;
- Gender-specific factors influencing adherence to ART have been noted in existing literature, with stigma often emerging as a significant obstacle for men, while women face challenges related to family responsibilities and trust in healthcare;
- Distance from healthcare facilities is a known determinant of poor adherence, with longer travel times associated with higher rates of treatment discontinuation.

### What this study adds

- Stigma is the most significant barrier to ART adherence among people living with HIV in Guinea, especially among male patients;
- Among female patients, low adherence is associated with being in treatment for more than one year, low trust in antiretroviral therapy, and not living with children;
- Patients who re-engaged in care after interruption were more likely to live farther from health facilities and to report lower adherence levels; trust in treatment and proximity to health services are positively associated with adherence across both sexes.
